# Upper limb motor assessment for stroke with force, muscle activation and interhemispheric balance indices based on sEMG and fNIRS

**DOI:** 10.3389/fneur.2024.1337230

**Published:** 2024-04-17

**Authors:** Sijia Ye, Liang Tao, Shuang Gong, Yehao Ma, Jiajia Wu, Wanyi Li, Jiliang Kang, Min Tang, Guokun Zuo, Changcheng Shi

**Affiliations:** ^1^Ningbo Institute of Materials Technology and Engineering, Chinese Academy of Sciences, Ningbo, China; ^2^University of Chinese Academy of Sciences, Beijing, China; ^3^Ningbo Cixi Institute of Biomedical Engineering, Ningbo, China; ^4^Department of Neurological Rehabilitation, Ningbo Rehabilitation Hospital, Ningbo, China; ^5^Robotics Institute, Ningbo University of Technology, Ningbo, China

**Keywords:** motor assessment, sEMG, fNIRS, stroke, elbow flexion

## Abstract

**Introduction:**

Upper limb rehabilitation assessment plays a pivotal role in the recovery process of stroke patients. The current clinical assessment tools often rely on subjective judgments of healthcare professionals. Some existing research studies have utilized physiological signals for quantitative assessments. However, most studies used single index to assess the motor functions of upper limb. The fusion of surface electromyography (sEMG) and functional near-infrared spectroscopy (fNIRS) presents an innovative approach, offering simultaneous insights into the central and peripheral nervous systems.

**Methods:**

We concurrently collected sEMG signals and brain hemodynamic signals during bilateral elbow flexion in 15 stroke patients with subacute and chronic stages and 15 healthy control subjects. The sEMG signals were analyzed to obtain muscle synergy based indexes including synergy stability index (*SSI*), closeness of individual vector (*C_V_*) and closeness of time profile (*C_T_*). The fNIRS signals were calculated to extract laterality index (*LI*).

**Results:**

The primary findings were that *C_V_*, *SSI* and *LI* in posterior motor cortex (PMC) and primary motor cortex (M1) on the affected hemisphere of stroke patients were significantly lower than those in the control group (*p* < 0.05). Moreover, *C_V_*, *SSI* and *LI* in PMC were also significantly different between affected and unaffected upper limb movements (*p* < 0.05). Furthermore, a linear regression model was used to predict the value of the Fugl-Meyer score of upper limb (FMul) (*R^2^* = 0.860, *p* < 0.001).

**Discussion:**

This study established a linear regression model using force, *C_V_*, and *LI* features to predict FMul scale values, which suggests that the combination of force, sEMG and fNIRS hold promise as a novel method for assessing stroke rehabilitation.

## 1 Introduction

Stroke is a neurological disorder characterized by vascular blockages, posing a severe threat to human health and life. Upper limb motor impairments are observed in 73 to 88% of first-time stroke survivors and 55 to 75% of chronic stroke patients (1). Therefore, a rational and effective strategy for assessing motor function is crucial for guiding the rehabilitation of stroke patients. In clinical practice, the Fugl-Meyer score of upper limb (FMul) is often used to evaluate upper limb motor function in stroke patients. However, the accuracy of this scale is typically dependent on the experience and subjective assessment of healthcare professionals. The quantitative assessment model that can elucidate the upper limb recovery process is necessary for better organizing rehabilitation strategies and enhancing overall recovery. Advanced imaging techniques have provided valuable information for diagnosis and functional prognosis (2).

In recent years, many studies have explored the use of various physiological signal indices for quantitative assessment of motor function in stroke patients, including surface electromyography (sEMG), electroencephalogram (EEG), and functional near-infrared spectroscopy (fNIRS), among others (3–5). However, many types of single evaluation index have better assessment performance over a specific time period, but could be less effective when covering long-term rehabilitation recovery process (6, 7). Therefore, most studies have strict requirements regarding the time of onset are imposed when selecting patients, often focusing on subacute periods within 3 weeks (4), subacute stage within 8 weeks (3) or chronic phases exceeding two months (5). Applying different assessment indices or parameters to patients with different onset times, while potentially increasing accuracy, also significantly complicates the assessment process, and it remains uncertain whether time-based categorization is the most reasonable approach.

Pino et al. (8) has proposed an assessment model based on structural reserve, the bimodal balance-recovery model, where structural reserve may be influenced by the severity of clinical damage, the integrity of brain regions, age, and other factors. Therefore, for a more precise assessment of patients with varying onset times and degrees of damage, a multi-dimensional evaluation is required. Both muscle synergy indices and indices of brain region activation are entry points for many studies to quantitatively assess stroke patients. A study of a set of upper extremity isometric strength tasks shows different muscle synergies in mild to severe stroke patients (9). Another study found that muscle synergy-related indices were positively correlated with function of neuromuscular control at the joint and task levels in stroke patients (5). These early studies strongly suggest that muscle synergy analysis based on sEMG may be a promising approach for assessing motor function in stroke patients. The fNIRS offers advantages such as non-invasiveness, mobility, resistance to motion and electromagnetic interference, high spatial resolution, and the ability to facilitate long-term monitoring, making it conducive to researching changes in cerebral hemodynamics during muscle contraction tasks (7, 10). fNIRS is also increasingly used to assess motor function or motor rehabilitation training effects (2, 11). Upper limb motor is the result of the coordination of the nervous system with the corresponding muscles. During the process of movement, there is varying degrees of information exchange between the brain and muscles, and motor dysfunction in stroke patients is due to problems in the brain’s control of muscle pathways (12, 13). The advantages of the fNIRS device make it very appropriate for the acquisition of cerebral hemodynamic changes during upper limb movement. Combined with the acquisition of sEMG signals, it can simultaneously obtain neurological information of the brain and muscles, which is helpful for analyzing the brain-muscle characteristic during movement. Although, few studies have analyzed sEMG signal and fNIRS signal of changes in cortical blood oxygen concentration simultaneously. Based on our knowledge, there is no previous research using them to the assessment of motor function in stroke. More recently, a significant positive correlation has been found between changes of muscle activation based on sEMG and cortical network dynamics based on fNIRS during isometric elbow contractions in healthy subjects (14). Therefore, combining fNIRS with sEMG likely provide a new perspective on stroke rehabilitation assessment.

In this study, sEMG and fNIRS were applied to extract effective information on peripheral muscle contraction and brain region activation, respectively, during specific motor tasks. Therefore, the aim of the present study was to explore muscle synergy indices and interhemispheric balance indices to co-assess upper limb motor function in stroke patients. We hypothesize that a linear regression model built from sEMG, fNIRS, and force indices collectively provide a robust assessment of upper limb motor function in patients.

## 2 Materials and methods

### 2.1 Subjects

This study encompassed two groups: stroke patients and healthy control group. The inclusion criteria for stroke patients were as follows: (1) first unilateral stroke; (2) possession of cognitive abilities required for task execution; (3) FMul score for the affected upper limb ranging from 10 to 66 out of a maximum score of 66; (4) time since stroke onset between 7 days and 2 years. Exclusion criteria included: (1) visual defects or achromatopsia; (2) presence of other neurological disorders such as Parkinson’s or Alzheimer’s disease. Following these inclusion and exclusion criteria, a total of 15 stroke patients (average age 56.27 ± 18.49 years, including 4 females) and 15 age-matched healthy control subjects (average age 52.20 ± 12.94 years, including 8 females) were recruited from the Rehabilitation Hospital of Ningbo. All participants provided written informed consent to participate in this study, which was approved by the Ethics Committee of the Rehabilitation Hospital of Ningbo in accordance with the Declaration of Helsinki (2023-16).

Furthermore, prior to the experiment, trained therapists assessed the motor function of the stroke patients using common clinical rating scales, including the FMul, Fugl-Meyer score of arm (FMarm) and Brunnstrom Scale (BS). The clinical characteristics of the stroke patients are presented in Table 1.

**Table 1:** Demographic and clinical characteristics of stroke patients.

Patient	Age	Sex	Lesion side	Type	Days after stroke	FMul	FMarm	BS
01	69	Male	Right	Ischemic	143	65	41	V
02	76	Female	Right	Ischemic	150	46	28	III
03	64	Male	Right	Hemorrhagic	121	66	42	V
04	36	Male	Left	Ischemic	26	41	30	IV
05	33	Male	Left	Ischemic	36	46	30	IV
06	32	Male	Right	Hemorrhagic	181	29	21	III
07	72	Male	Right	Hemorrhagic	126	16	13	II
08	36	Female	Right	Ischemic	52	26	16	III
09	86	Male	Right	Hemorrhagic	36	49	29	IV
10	65	Male	Right	Hemorrhagic	573	27	13	III
11	30	Male	Left	Hemorrhagic	197	17	15	III
12	73	Female	Right	Ischemic	12	65	41	VI
13	60	Male	Left	Ischemic	32	64	40	V
14	52	Male	Left	Hemorrhagic	12	34	18	IV
15	60	Female	Left	Ischemic	43	29	23	III

### 2.2 Motor task paradigm

**Figure 1:**
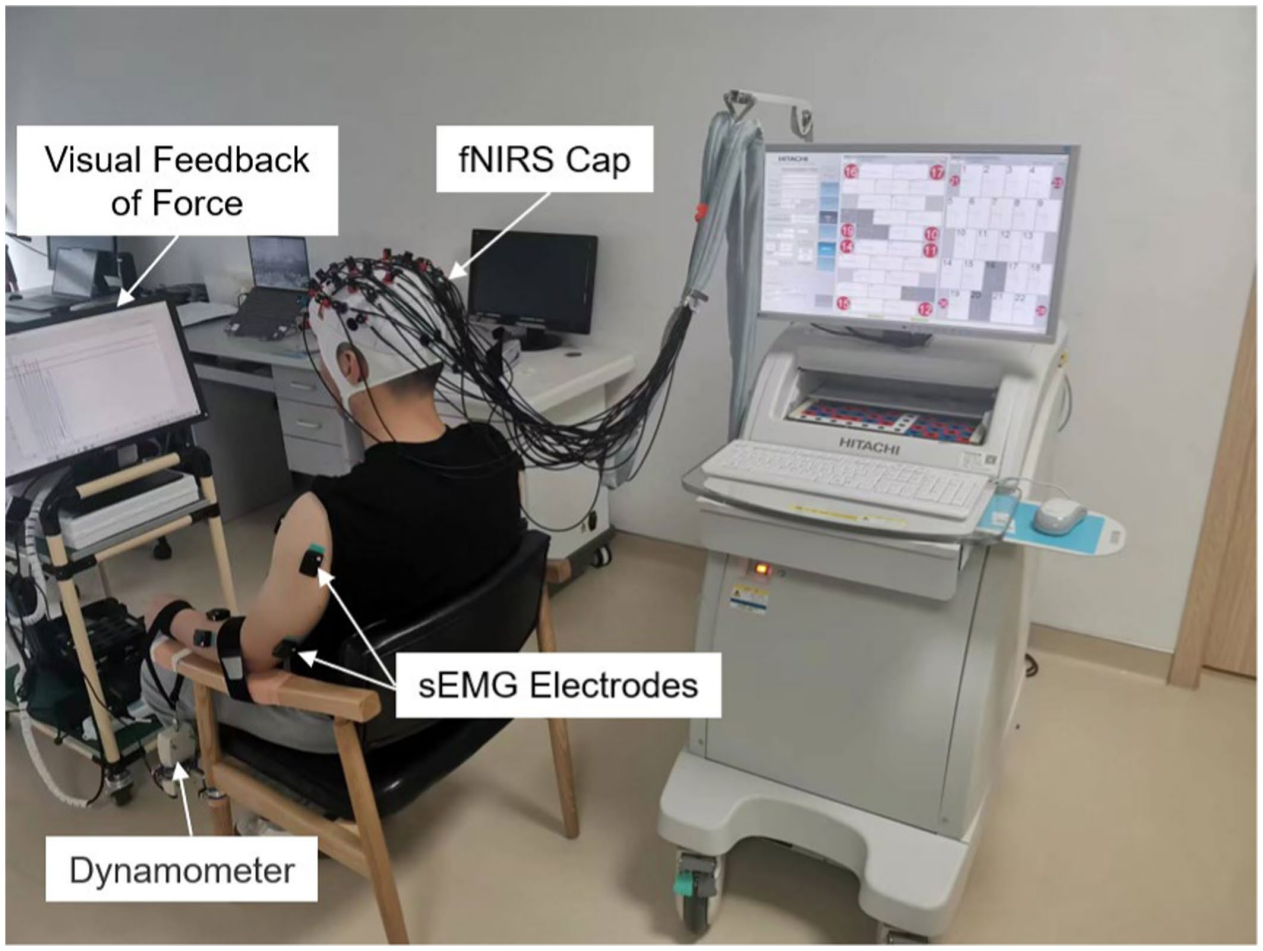
Experimental scenario.

Referring to a similar paradigm of using fNIRS to probe brain function in patients (7), participants were seated comfortably in a designated chair, facing a computer monitor, with both arms resting on the chair’s armrests, palms facing upward, as depicted in Figure 1. The arm to be moved during the task was securely fastened to the armrest using a 5 cm wide, slightly elastic strap to prevent the elbow from lifting off the armrest during elbow flexion. Additionally, a non-extendable strap connected to a dynamometer was secured around the wrist of the arm to be moved, with the dynamometer fixed to the chair’s leg. The force signal is recorded at a sampling frequency of 10 Hz and synchronized with the sEMG equipment. The force sensor is used for real-time feedback of actual force magnitude to the subjects during paradigm tasks and for subsequent sEMG data segmentation and screening of valid data.

Before the formal commencement of the task, participants were instructed to avoid unnecessary movements and thoughts once the experiment began. Each participant also had a prior opportunity to familiarize themselves with the task paradigm to ensure a correct understanding and execution of the instructed actions. Initially, participants performed three maximal voluntary contraction (MVC) of elbow flexion, and 30% of the mean of these three values was set as the target force for the actual task. After participants were adequately prepared, the equipment provided the “raise a hand “command, and participants performed 15 s of intermittent isometric muscle contractions of the forearm on the designated arm, guided by an auditory cue with a 0.5 Hz frequency and 2/4 -beat, followed by a 25-s rest period after the equipment issued the “relax” command. Each block comprised six trials. During the task, the monitor in front of the participants displayed a target range of ±5% of the target force, providing feedback on the real-time actual force output. Participants were required to control the force within the target range as accurately as possible. There were two blocks in total, with the first block being completed by the participants’ unaffected arm (the dominant hand for the control group) and the second block by the participants’ affected arm (the non-dominant hand for the control group), with at least a 5-min rest period between the two blocks.

### 2.3 Data acquisition

#### 2.3.1 sEMG and force data acquisition

In this experiment, the Trigno Wireless EMG System (Delsys Inc., Boston, MA, United States) was used to capture sEMG signals. A dynamometer (SH-100 N, HANDPI company, China, precision of 0.01 N) was used to capture force signals. The sEMG signals and force signals were synchronized using data analysis software and sampled at frequency of 1,925.9 Hz and 10 Hz, respectively. To facilitate subsequent data segmentation, the sEMG data with dynamometer data were synchronized sampled at 10 Hz, ensuring that each sEMG signal was marked with the corresponding dynamometer data at each acquisition point. The recorded muscles included the bilateral anterior deltoid (DA), posterior deltoid (DP), biceps brachii (BI), triceps brachii (TI), and brachioradialis (BIO). Before attaching the sensors, the muscles corresponding to both upper limbs of the participants were wiped twice with 75% alcohol to ensure more accurate signal acquisition. Subsequently, accordance with the guidelines of SENIAM and a therapist, the sensors were affixed to the belly of the respective muscles (15).

#### 2.3.2 Brain hemodynamic data acquisition

**Figure 2:**
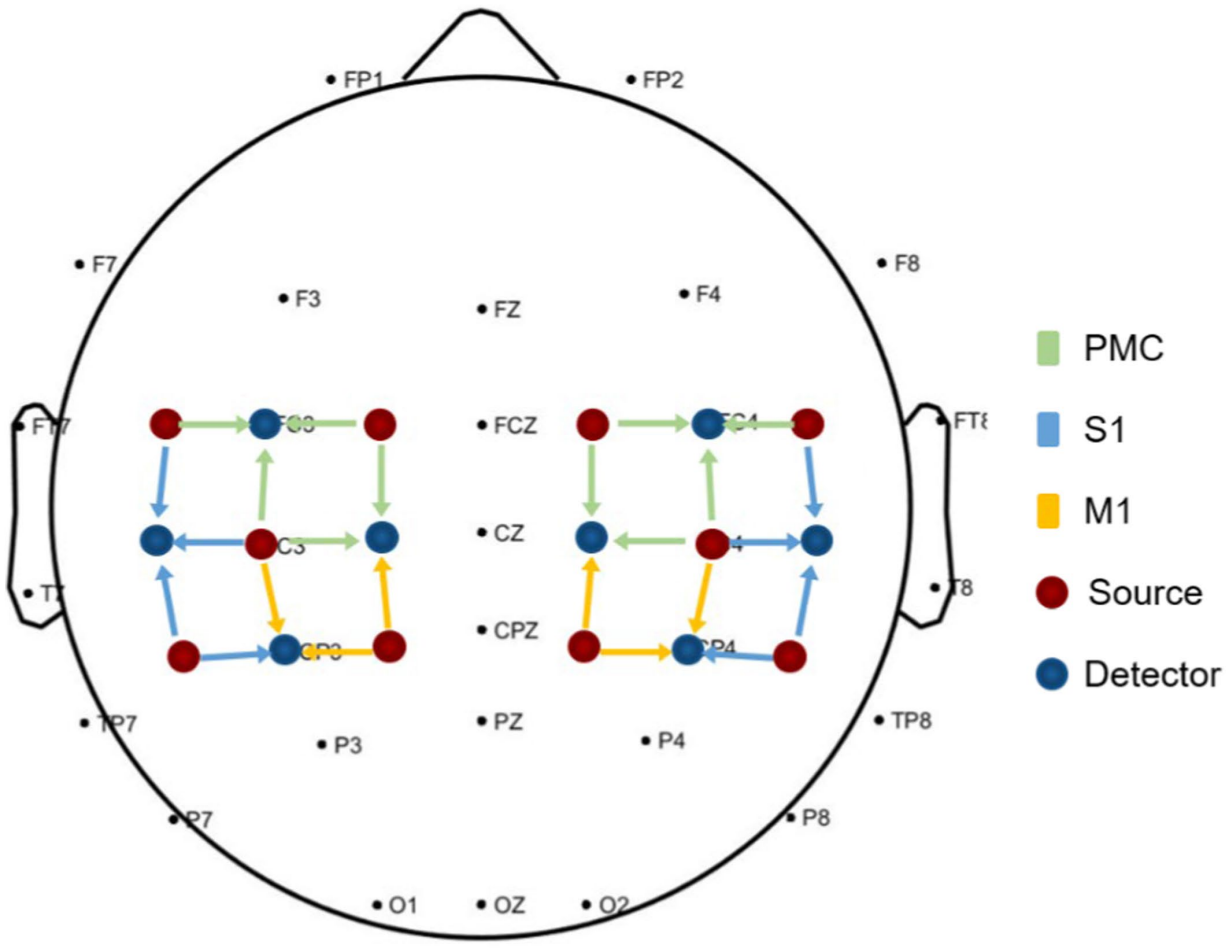
The regions of interest in fNIRS acquisition.

This study utilized Optical Topography system with wavelengths of 830 nm and 704 nm (ETG-4100, Hitachi Medical Co., Japan) to collect data on cerebral blood flow changes at a frequency of 10 Hz. Two sets of 3 × 3 probe arrays were employed, with each probe array consisting of 5 sources and 4 detectors, forming measurement channels (for a total of 24 channels). The placement of the probes followed the international 10/20 electrode placement system, with a spacing of 30 mm between sources and detectors. These channels symmetrically covered the bilateral pre-motor cortex (PMC), primary motor cortex (M1), and somatosensory cortex (S1) on both sides, as illustrated in Figure 2. To facilitate data analysis and result presentation, the right hemisphere was defined as the affected hemisphere, and the left and right hemispheric channels of the patients whose lesions were originally located in the left hemisphere were switched.

### 2.4 sEMG data preprocess and analysis

#### 2.4.1 sEMG data preprocess

The collected raw sEMG signals underwent a series of preprocessing steps. Initially, a fourth-order bandpass Butterworth filter (20-450 Hz) was applied to the signals to eliminate any noise (16). Subsequently, the signals were detrended and rectified to obtain the full-wave rectified sEMG signal. To calculate the envelope of the sEMG signal, a fourth-order low-pass Butterworth filter with a cutoff frequency of 5 Hz was employed (16, 17). Subsequent segmentation of the sEMG signal window was performed on the basis of the change in signal acquired by the dynamometer. Complete sEMG data for a single hand lift from two time periods before dynamometer readings rise to return to baseline. Among them, data with a single lifting cycle of more than 3 s and force readings that deviate from the target value by more than 15% will be excluded. Finally, the segmented data were normalized using both maximum and minimum values and time normalization to facilitate the subsequent extraction of muscle synergy.

#### 2.4.2 Muscle synergy extraction

For the computation of muscle synergy, the Multivariate Curve Resolution Alternating Least Squares (MCR-ALS) algorithm was employed (17). Because compared with other commonly used muscle synergy algorithms, MCR-ALS solves the problem that NMF and ICA algorithms tend to fall into local optimal solutions and lead to poor reproducibility by finding pure variables based on the SMMA algorithm (18). It also improves the problem that SVD-NMF and FA can hardly ensure the natural properties of muscle synergy by alternating least squares (ALS) and imposing non-negative constraints to ensure that the decomposition results are physically meaningful. Therefore, MCR-ALS has better consistency and internal stability in the context of stroke patients with motor impairments (19). The algorithm was applied to the sEMG signals recorded during upper limb movements on both arms for all participants. In carrying out the calculation of indices, it is also necessary to obtain baseline synergies, denoted as *V_B_* and *T_B_*. This value was also calculated from this algorithm, using data from the healthy control group separately by dominant hand and non-dominant hand and averaged overlay. The synergy decomposition process can be formulated as follows:


$$X_{p\times q}=V_{p\times n}T_{n\times q}+E_{p\times q}$$

In this equation, *E* represents the residual, *V* includes the base vectors representing the synergy space, and *T* is the matrix of time profiles. Parameters *p*, *q*, and *n* correspond to the number of sEMG channels, the time-normalized number of samples (which is 50 in this case), and the number of synergies, respectively.

To determine the most suitable number of synergies, variance accounted for (*VAF*) was utilized to evaluate the goodness of fit for different numbers of synergies (17, 20). The *VAF* was calculated using the following equation:


$$VAF=1-\left(\frac{||X-M||{\;}^{2}}{||X-\mathrm{mean}\left(X\right)||{\;}^{2}}\right)$$

Here, *M* represents the reconstructed sEMG matrix, and the “mean” operator generates a matrix of the same size as *X*, with columns representing the mean values of the corresponding columns in *X*. In this study, when more than half of the participants in both groups had *VAF* values exceeding 80%, *n* was defined as the minimum value (5). Consequently, a single synergy (*n* = 1) was sufficient to meet this criterion, and thus, a single synergy was utilized for subsequent indices calculations.

#### 2.4.3 Index of muscle synergy

In order to find better indices of muscle synergy that could potentially contribute to the quantitative assessment, we selected three indices from three areas, namely *SSI*, *C_V_* and *C_T_*. Where *SSI* was used to assess the stability of muscle synergy between trials in the same subject; *C_V_* was used to assess the proximity of the degree of activation of each muscle in the patient to that of the healthy control; and *C_T_* was used to assess the similarity of muscle activation on the timeline between the patient and the healthy control. To quantitatively assess the outcomes of muscle synergy decomposition for different participants, several indices were utilized for evaluation. These indices encompass the *SSI* (21), *C_V_* (5) and *C_T_* (22). Below, you will find the key formulas for these three indices:


$$SSI=\frac{1}{K}\left[\frac{2}{i\left(i-1\right)}\overset{p}{\underset{l\neq q}{\sum }}r\left(V_{l},V_{q}\right)\right]$$

Here, *i* denotes the total number of unilateral upper limb raising occurrences by a participant. *K* represents the number of synergies. *V_l_* and *V_q_* represent the base vectors for the *l* and *q* instances of limb raising. *r* denotes the Pearson correlation coefficient.


$$C_{V}\left(i\right)=\frac{V\left(i\right)\cdot V_{B}}{\left(\left|V\left(i\right)\right|\cdot \left|V_{B}\right|\right)}$$

$$C_{T}\left(i\right)=\frac{\left|I\left(\tau \right)\right|}{\sqrt{\sum_{m=1}^{50}T^{2}\left(m\right)\cdot \sum_{m=1}^{50}{T_{B}}^{2}\left(m\right)}}$$

Since this study employed a single synergy (*n* = 1), the presented formulas are simplified versions that do not include synergy number variables. In these formulas, *i* denotes the number of times the limb was raised, while *V* and *V_B_* represent the muscle synergy’s base vectors for individual instances and the mean baseline synergy from all control subjects, respectively. *T* and *T_B_* similarly represent the corresponding coefficient time profiles. *I*(*τ*) is the circular cross-correlation function with a time-lag between the two profiles. The range of *C_V_* and *C_T_* falls between 0 and 1, with 1 indicating the highest degree of similarity.

### 2.5 Brain hemodynamic data preprocess and analysis

#### 2.5.1 Brain hemodynamic data preprocess

Since oxygenated hemoglobin (HbO) signal data shows more pronounced activation compared to deoxygenated hemoglobin (HbR) signal data (23, 24), all subsequent analyses were conducted exclusively on HbO signals. After obtaining the raw optical intensity signals from the equipment, preprocessing was carried out using data analysis software and codes from the open-source Homer2 toolbox (25). The preprocessing steps encompassed the following: (1) Transformation of the raw optical intensity signals into optical density. (2) Removal of motion artifacts using a kurtosis-based wavelet algorithm (k = 3.3) (26). (3) Application of a third-order bandpass Butterworth filter with a frequency band of 0.01–0.08 Hz to eliminate unwanted physiological signals or environmental noise. (4) Conversion of optical density data into hemoglobin concentration data using the modified Beer–Lambert law (27). (5) Computation of *β* coefficients for all 24 channels via the General Linear Model (GLM) codes from NIRS-KIT (28) and NIRS-SPM (29), which represented the degree of neural activation during each activity (30).

#### 2.5.2 Brain hemodynamic data preprocess

The Laterality Index (*LI*) is regarded as a potential index in stroke functional neuroimaging research, enabling the assessment of hemispheric activation balance during upper limb movement, and was used in fNIRS study (7, 13). *LI* is calculated based on *β* values and defined as follows:


$$LI_{\mathrm{region}}=\frac{\beta_{C-\mathrm{region}}-\beta_{I-\mathrm{region}}}{\beta_{C-\mathrm{region}}+\beta_{I-\mathrm{region}}}$$

Here, *β_C-region_* represents the average *β* value for channels in the contralateral brain region. *β_I-region_* represents the average *β* value for channels in the ipsilateral brain region. The “region” includes PMC, M1, and S1, which are the three regions of interest, that is, we calculated the *LI* values for PMC, M1, and S1. A higher *LI* value indicates a greater degree of contralateral activation for healthy or contralesional activation for patients, while a smaller or negative *LI* value indicates more pronounced ipsilateral activation for healthy or ipsilesional activation for patients.

### 2.6 Regression analysis

This study employed a multiple linear regression approach to examine the correlations between various indices or factors and clinical scale scores. Recognizing that the indices of the unaffected arms of the patients may also provide insights into motor function, the indices *LI_region_* (*LI_PMC_*, *LI_M1_* and *LI_S1_*), *SSI*, *C_V_* and *C_T_* considered for regression analysis included both the unaffected and affected sides. Additionally, we incorporated the ratios of these four indices between the unaffected and affected arms (*LI_region-r_*, *SSI_r_*, *C_V-r_* and *C_T-r_*). It has been suggested in prior studies that the correlation between patient motor function scale scores and various factors such as age and time since the onset of the condition is significant (2). Therefore, in addition to the aforementioned indices, we also incorporated age, time since onset, and the ratio of maximum voluntary contraction force between the affected and unaffected hands *F_r_* into our analysis. To identify the most influential factors for predicting clinical scale scores, this study systematically evaluated all combinations of the 21 predictor variables to determine the four variables that yielded the highest goodness of fit in the multiple linear regression equation. Subsequently, we employed these parameters separately to establish multiple linear regression equations with the FMul scale scores. Finally, scatter plots of fitted and actual scores for healthy individuals and patients were constructed.

### 2.7 Statistical analyses

Statistical analyses of the various indices were conducted using SPSS (V.27, IBM, United States) and R (4.3.3, Lucent, United States). Initially, the Shapiro–Wilk test and Levene’s test were used to assess the normality and homoscedasticity of the indices data. If both assumptions were met, independent-sample t-tests were employed to analyze significant differences in indices between patients and the control group, paired-sample t-tests were used to analyze differences between indices of both upper limbs, and two-way analysis of variance (ANOVA) was utilized to examine the interaction effects of group (cross-side, namely the pool of affected arm and unaffected arm or non-dominant hand and dominant hand) and arm (cross-group, namely the pool of patients and control subjects). If the assumptions were not met, non-parametric tests such as the Mann–Whitney U test, Wilcoxon signed-rank test, and Scheirer-Ray-Hare test were applied. For the multiple linear regression models, F-tests were used to assess the overall significance of the regression equations, while t-tests were used to evaluate the significance of individual coefficients. A two-tailed analysis was performed, and the level of statistical significance was set at *p* < 0.05.

## 3 Results

### 3.1 Muscle synergy activation mode

**Figure 3:**
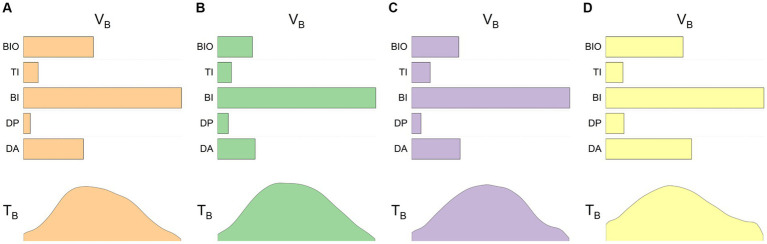
Baseline synergies in control subjects and patients. The four sets of images represent the averaged data for four different conditions: **(A)** control subjects’ dominant arm, **(B)** control subjects’ non-dominant arm, **(C)** stroke patients’ unaffected arm, and **(D)** stroke patients’ affected arm. The upper half of each image shows the base vectors in the synergy space, while the lower half provides the corresponding time profile information.

Figure 3 illustrates the average muscle synergy patterns for the dominant and non-dominant arms of the control group and the unaffected and affected arms of the patients. From the images, it can be observed that the synergy patterns for the control group’s dominant and non-dominant hands are quite similar, as are those for the unaffected arms of the patients. However, the patient’s affected side, while primarily activating the biceps brachii like the healthy side, also exhibits more activation of the anterior deltoid and brachioradialis muscles. Although there are slight differences in the details of the time profile vectors, the overall patterns are similar.

### 3.2 Index of muscle synergy

**Figure 4:**
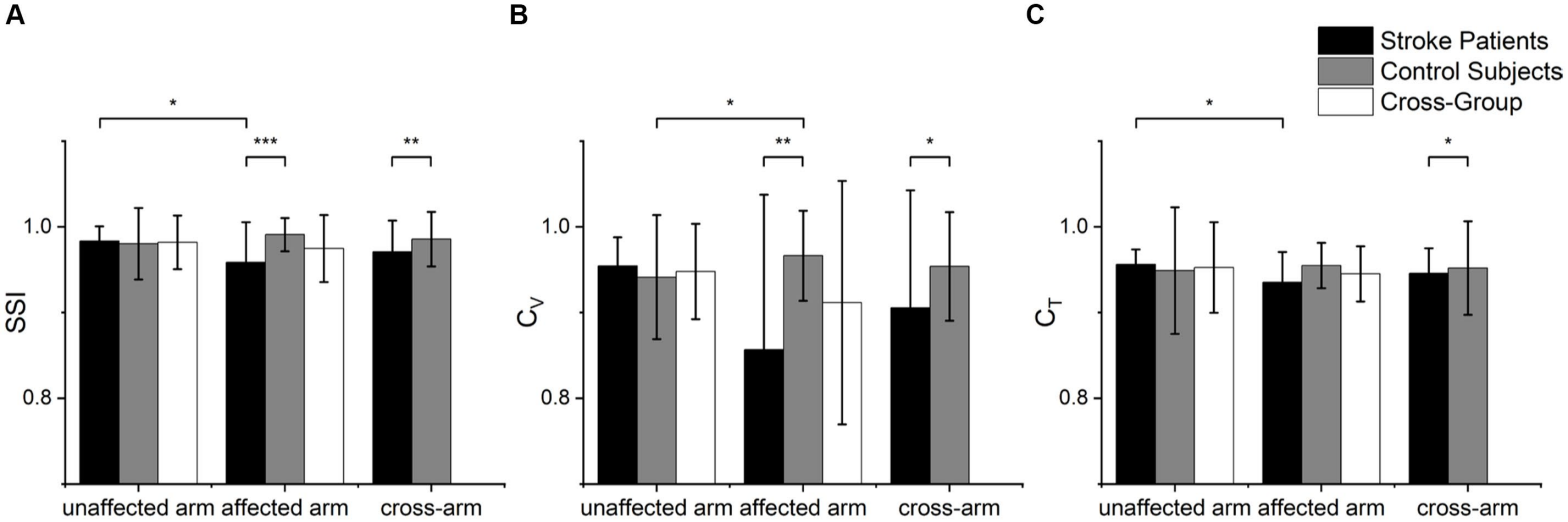
Statistical analysis of muscle synergy indices of **(A)** *SSI*, **(B)** *C_V_* and **(C)** *C_T_*. Scheirer-Ray-Hare tests were used to analyze group factors (cross-arm) and arm factors (cross-group), while Mann–Whitney U tests were used to examine significant differences in various index values between different groups. Wilcoxon signed-rank tests were employed to analyze index values within different sides (*p < 0.05, **p < 0.01, ***p < 0.001).

The statistical analysis results of the *SSI*, *C_V_* and *C_T_* indices for both arms of the patients and healthy controls are depicted in Figure 4. Significant differences were found between the patient’s affected arm and the control group’s non-dominant hand in terms of *SSI* (*p* < 0.001) and *C_V_* (*p* = 0.007). Differences were also observed in the indices *SSI* (*p* = 0.036) and *C_T_* (*p* = 0.023) between the patient’s unaffected and affected sides. Although there was no significant difference in the *C_V_* index between the patient’s unaffected and affected arms (*p* > 0.05), their means were notably different, possibly due to the non-parametric test leading to a loss of information and reduced test efficiency. There was a difference in the *C_V_* index between the control group’s healthy and affected arms (*p* = 0.031). Furthermore, all three indices, *SSI* (*p* = 0.005), *C_V_* (*p* = 0.036), and *C_T_* (*p* = 0.013), exhibited significant differences in the cross-group.

### 3.3 Cortical activation patterns

**Figure 5:**
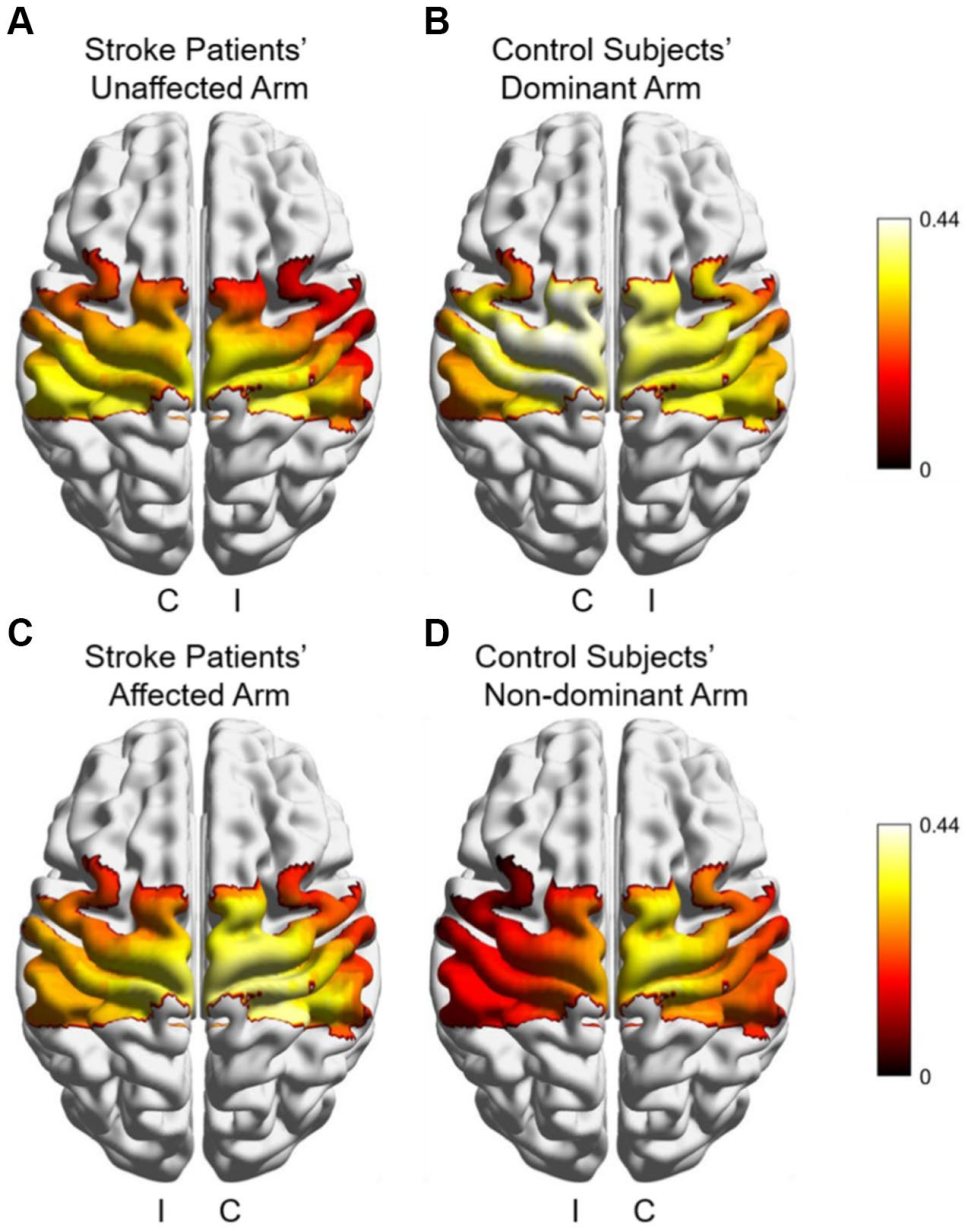
Cortical activation patterns with respect to HbO of *β* during different arms and groups in subjects **(A)** Stroke patient’s unaffected arm; **(B)** controls’ dominant arm; **(C)** patient’s affected arm, and **(D)** controls’ non-dominant arm. I: ipsilateral brain region, C: contralateral brain region.

Figure 5 shows cortical activation patterns with respect to HbO of *β*, which was created by BrainNet Viewer (31). Overall, activation appeared to be stronger in contralateral brain regions in all groups and conditions. The healthy control had the highest overall degree of activation during dominant hand movements, while its ipsilateral brain regions had lower activation during non-dominant hand movements compared to all other groups and conditions. Using the Wilcoxon signed-rank test to compare activation between the unaffected and affected arm of the patients revealed no significant differences in any of the brain regions. In contrast, there were two ipsilateral brain regions in the control group that had significantly higher activation during dominant hand movements than non-dominant hand movements, IM1 (*p* = 0.003) and IPMC (*p* < 0.001). No significant differences were found when comparing patients and controls using the Mann–Whitney U test. These results suggest that healthy controls had greater differences in activation of brain regions during bilateral upper limb movements, whereas patients had more similar activation of each brain region during bilateral upper limb movements.

### 3.4 Index of brain lateralization

**Figure 6:**
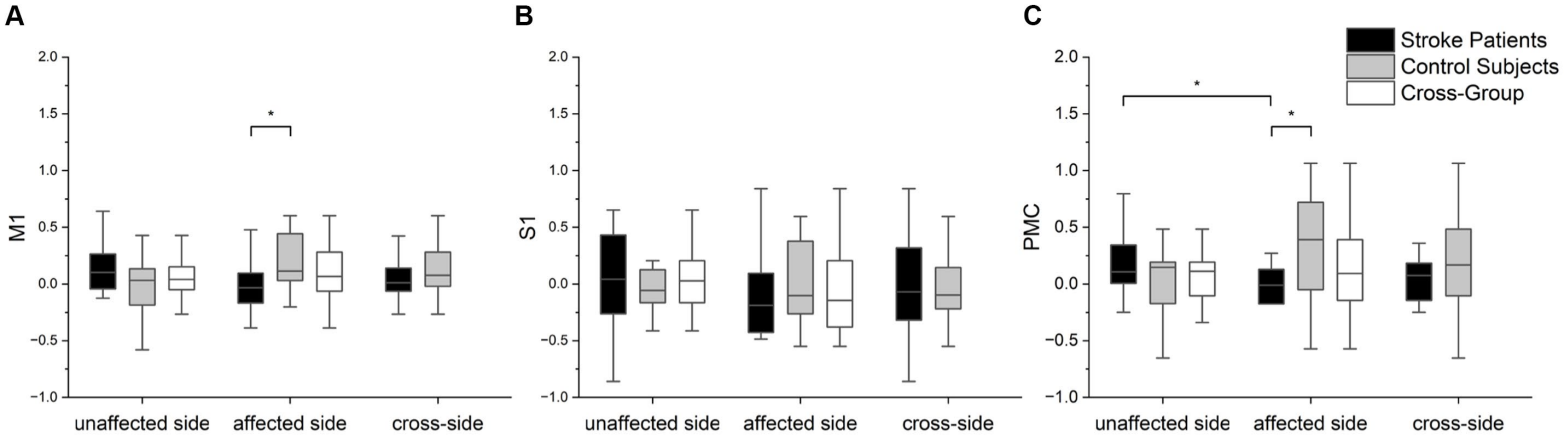
The statistical analysis of three brain regions of interest (*LI* values) between the two groups and arms: **(A)** M1, **(B)** S1, and **(C)** PMC. Scheirer-Ray-Hare tests were performed with the factor of group (cross-task) and factor of group (cross-group). Mann–Whitney U tests were employed to assess the significance of differences in *LI* values among different groups for each brain region. Furthermore, we used Wilcoxon signed-rank tests to statistically analyze differences in *LI* values within groups for different arms. In the box plots within the figure, the lower, middle (bold), and upper horizontal lines represent the 25th, 50th (median), and 75th percentiles, respectively. Significance levels are indicated as *p < 0.05.

The statistical analysis results of *LI* in different brain regions for both arms of the patients and both arms of the control group are presented in Figure 6. Significant differences were observed in the M1 (*p* = 0.044) and PMC (*p* = 0.026) brain regions between the patient’s affected arm and the control group. Additionally, differences were noted in the PMC brain region concerning the patient’s unaffected and affected hemisphere (*p* = 0.008). However, no differences were observed in the S1 brain region. These results suggest that changes in PMC brain region lateralization may better differentiate between the patient’s unaffected and affected arms or between the patient and the control group in terms of motor function.

### 3.5 Regression analysis

**Figure 7:**
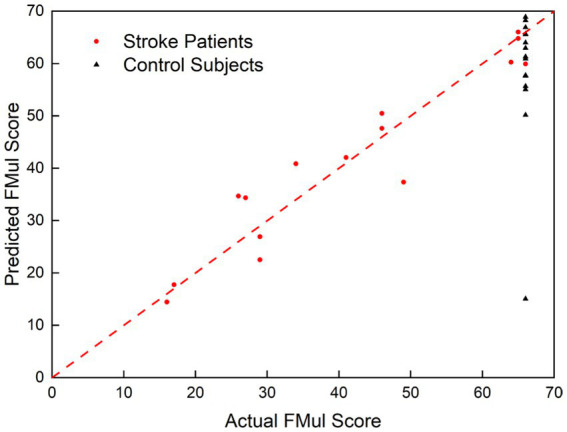
Scatterplots showing the relations between the actual and predicted FMul scales.

We conducted regression analysis using the previously mentioned method, exploring all combinations of factors. Among them, the regression model with the highest goodness of fit to the FMul included the following factors: *C_V-af_* (*af* means affected side), *LI_PMC-un_* (un means unaffected side,), *F_r_* and *C_V-r_*, which the adjusted *R^2^* value stands at 0.860. Correlation analysis revealed no significant correlations between any two of the three predictive factors: *C_V-af_*, *F_r_*, and *LI_PMC-un_*. Table 2 presents a comprehensive list of all model parameters, featuring B values, *β* coefficients, and *p*-values. The regression model showed that the predictive factors *C_V-r_*, Fr, and *LI_PMC-un_* could all predict upper limb motor function in patients and healthy controls (Figure 7), and the prediction model was significant (*p* < 0.001). Nine of the fitted score values for healthy people were greater than 60. The regression equation was as follows:


$$\begin{array}{l} \mathrm{FMul}=-200.24+133.49\cdot C_{V-af}+30.33\cdot LI_{PMC-un}+\\ 45.95\cdot F_{r}+83.70\cdot C_{V-r} \end{array}$$

where *C_V-af_* represented the *C_V_* of affected side, *C_V-r_* represented the *C_V_* on the unaffected arm to that on the affected side, *LI_PMC-un_* represented the *LI* of unaffected arm in PMC region and *F_r_* represented the ratio of MVC force on the unaffected arm to that on the affected side.

**Table 2:** Multiple linear regression model.

Independent variable	Multi-linear regression			Simple linear regression	
	B	β	*p* value	*R*^2^	*p* value
Constant	−200.24		0.005		
C_V-af_	133.49	1.36	0.007	0.095	0.263
LI_PMC-un_	30.33	1.34	<0.001	0.270	0.047
F_r_	45.95	0.82	<0.001	0.375	0.015
C_V-r_	83.70	2.23	0.001	0.105	0.240

The goodness of fit and significance of the simple linear regression of each of the above parameters with the scale FMul are shown in Table 2. The goodness of fit for each parameter was not high, with the highest being *F_r_* at 0.375.

## 4 Discussion

This study aimed to explore muscle synergy indices and interhemispheric balance indices to co-assess upper limb motor function in stroke patients. When compared to the healthy control group, stroke patients exhibited significantly reduced sEMG index *C_V_* on the affected arm and decreased cerebral blood flow *LI* values in the region of PMC. Moreover, during movements on the affected side, stroke patients demonstrated lower *LI* values in the PMC compared to movements on the unaffected side. These results indicate abnormal muscle synergy patterns and altered activation of specific brain regions in stroke patients, which can jointly predict clinical scale scores and serve as assessment indices for stroke.

All three indices of muscle synergy, including *SSI* (stability), *C_V_* (closeness), and *C_T_* (time), indicate that the muscle synergy pattern on the affected arm of stroke patients is weaker than that of healthy individuals. In our study, we attempted three different indices: *SSI*, *C_V_* and *C_T_*. *SSI* are often used to assess the stability of muscle synergy between different trials in the same patient. Studies have shown that assessing the stability of the synergy structure vectors *V* across different trials can be used to evaluate clinical motor function and is positively correlated with clinical assessment scores of upper limb motor function (5, 32). However, the findings showed a low correlation with clinical scores, and we speculate that it may be that some patients are also able to have more stable control of their muscles with diligent rehabilitation. *C_V_* and *C_T_* indices assess the closeness between muscle synergy decomposition vectors *V* and time-profile vectors *T* from the perspective of muscle synergy (22, 33, 34). The statistical results show that both the stability and closeness of the muscle synergy structure vectors *V* in stroke patients are significantly different from those in the control group, but there is no difference in the time profile vectors *T*. The time course of the patient’s performance in the index value *C_T_* was not significantly different from that of healthy individuals, suggesting that this is not a good indicator to differentiate the patient’s motor function. The *C_V_* values are a good response not only to whether the patient’s synergistic pattern of the muscles during motion is closer to that of a healthy individual, but also to the ability to efficiently carry out the muscle utilization, and more severe co-movement is associated with lower clinical scores, and therefore has a better performance in the evaluation model has a better performance. The changes in these muscle synergy vectors not only reveal damage to individual muscle control but also reflect neural reorganization in the brain, which is an important index of motor function recovery after stroke (35).

In the study of brain hemodynamic data, the results showed significant ipsilateral activation of both M1 and PMC during dominant hand compared to non-dominant hand in the control group, and significant differences in *LI* indices were observed in the PMC and regions between stroke patients and healthy controls. GLM has been shown to be an effective method for examining hemodynamic responses in fNIRS analysis (36–38). In this study, we considered *β* values as a measure of cortical activation, with higher β values indicating higher activation in the brain regions corresponding to this channel. The activation of ipsilateral M1 and PMC brain regions was significantly higher in the control group during dominant hand movements than during non-dominant hand ones, which may be due to the degree of brain activation related to the redistribution of brain resources (39). In order to achieve more precise force control, the brain regions controlling unilateral movements will be more active during sharp hand movements, whereas skillful reallocation of brain resources is difficult to achieve with non-sharp hand movements. However, the activation of various brain regions during movement on the affected arm is altered in stroke patients, and the recovery of motor function following stroke is closely related to the reconstruction of the motor system in both the contralateral and ipsilateral hemispheres (40). *LI*, as a quantitative method for functional neuroimaging studies in stroke recovery, describes the contrast in activation levels between contralateral and ipsilateral hemisphere (7, 13). Combined with *β* values as a measure of activation, *LI* is suitable for assessing patient motor function. We evaluated *LI* during movements on the unaffected and affected hemisphere of stroke patients and found significantly lower *LI* indices in the M1 and PMC regions during affected arm movements compared to non-dominant hand movements in healthy individuals. The M1 plays a key role in improving control of distal arm muscles during movement (41), and the PMC is also involved in the recovery of motor function in chronic stroke patients (42). The decrease in *LI* in these two brain regions suggests impairment of upper limb function corresponding to these regions (7, 43), resulting in increased activation in the ipsilateral hemisphere during affected arm movements, indicative of a compensatory mode within the recovery model. Interestingly, we also observed that the *LI* values in the PMC during movements on the unaffected arm of stroke patients were significantly higher than the *LI* values during affected arm movements. According to Figure 6, the *LI* values in the PMC during movements on the healthy arm of stroke patients were slightly higher than those in the healthy control group. This may be explained by another recovery model—interhemispheric inhibition.

*C_V_*, *LI*, and *F_r_* as quantitative methods for functional neuroimaging studies in stroke recovery, can predict upper limb motor function in patients. This provides new insights for guiding stroke patient rehabilitation, as well as assessing and predicting the recovery status of patients. In this study, we attempted to fit linear regression equations with various features, including sEMG signals, cerebral blood flow, and force, to FMul scores. The results showed that the equation with the highest goodness of fit (*R*^2^ = 0.860) included information from sEMG, cerebral blood flow, and force, indicating that the combination of physiological signals reflected by the brain and muscles during the movement process can better assess patient motor function. This assessment model contains *F_r_*, *C_V_*, and *LI*, which corresponds to the patient’s residual force magnitude, synergistic motor function of the muscles, and hemispheric lateralization of cerebral activation, which is well adapted to the two important indices of residual motion function, and inter-hemispheric cerebral balance, proposed by the bimodal balance-recovery model. Age, days after stroke, and other indices were poorly assessed in this study for stroke patients, which may mean that these factors are weakly related to the assessment of motor function in stroke patients, or it may be that these factors are already expressed by *F_r_*, *C_V_*, and *LI*. However, the goodness of fit for simple linear regression equations with individual features and FMul scores was generally low, suggesting a relatively weak linear correlation between single indices and the scores. A single index may be useful for evaluating a specific range of stroke patients, but it will be much less stable when applied to a wider range of situations, or it may be understood that an increase in the value of this index does not imply an increase in the patient’s actual motor function. This result is consistent with many longitudinal studies, where the *LI* in stroke patients tends to increase initially after stroke, then decrease around two months to three months (7), with some studies showing a significant average decrease in *LI* at approximately one week after stroke, followed by an increase after three to six months (44). Some studies have not found enough significant difference in *LI* (11, 45). Similar patterns have been observed for motor evoked potentials (MEPs) and sEMG (46, 47). At this point, the advantage of co-assessment of multiple indices comes into play. Our model improves the stability and accuracy of the model by extracting the force signal, sEMG signal, and fNIRS signal and by building a multivariate linear assessment model, assigning different weights to each index, so as to minimize the problem that a single feature fails in some situations. In addition, we applied the model to a healthy control group and found that most of the fitting scores were above 60. It was further verified that the physiological indices used in the model had practical significance, meaning that the better-fit scores showed that the patient’s indices were closer to those of a healthy person. In conclusion, the combination of physiological indices in this quantitative assessment model can be well used to predict the score values of the clinical scale FMul. Our next step is to continue to track the patient’s recovery and to try other indices in order to establish a more stable and accurate model.

There are some limitations to this study. Firstly, the sample size was relatively small. To make the analysis results more convincing, future studies should recruit a larger number of stroke patients. Secondly, the paradigm design of this study required patients to have a certain level of motor function to resist gravity and perform lifting movements. Therefore, this study lacked severely impaired patients. Additionally, due to some patients not being able to perform isolated elbow flexion movements effectively, there may be variations in the force generation method, which could potentially influence the results. From an assessment modeling perspective, manual segmentation of the sEMG signal data is required before calculating the muscle synergy metric values, which makes the calculation of the assessment results take some time. Lack of analysis of DeoxyHb change in fNIRS signals, which may result in the absence of valid information. The indices and their algorithms used for modeling in this study are basic, and better indices or algorithms may exist.

## 5 Conclusion

To the best of our knowledge, this is the first study to combine force, sEMG, and fNIRS features during elbow flexion for stroke assessment. Furthermore, we included both subacute and chronic phase patients in the study. The results indicate that stroke patients exhibit significantly lower muscle synergy structure vector indices (*SSI*, *C_V_*) and reduced *LI* values in the PMC and M1 regions compared to healthy controls, reflecting potential mechanisms underlying functional impairment in stroke patients. Moreover, we established a linear regression model using *C_V_* and *LI* features to predict FMul scale values. *F_r_*, *C_V_*, and *LI* hold promise as beneficial biomarkers during the stroke motor recovery process.

## Data availability statement

The raw data supporting the conclusions of this article will be made available by the authors, without undue reservation.

## Ethics statement

The studies involving humans were approved by Ethics Committee of the Rehabilitation Hospital of Ningbo. The studies were conducted in accordance with the local legislation and institutional requirements. The participants provided their written informed consent to participate in this study.

## Author contributions

SY: Conceptualization, Data curation, Formal analysis, Methodology, Software, Writing – original draft. LT: Conceptualization, Methodology, Writing – original draft. SG: Investigation, Methodology, Writing – original draft. YM: Methodology, Writing – original draft. JW: Software, Writing – original draft. WL: Project administration, Writing – original draft. JK: Investigation, Writing – original draft. MT: Conceptualization, Resources, Supervision, Writing – review & editing. GZ: Conceptualization, Funding acquisition, Supervision, Writing – review & editing. CS: Conceptualization, Funding acquisition, Methodology, Project administration, Supervision, Writing – review & editing.
